# Choice of PD-L1 immunohistochemistry assay influences clinical eligibility for gastric cancer immunotherapy

**DOI:** 10.1007/s10120-022-01301-0

**Published:** 2022-06-04

**Authors:** Joe Yeong, Huey Yew Jeffrey Lum, Chong Boon Teo, Benjamin Kye Jyn Tan, Yiong Huak Chan, Ryan Yong Kiat Tay, Joan Rou-En Choo, Anand D. Jeyasekharan, Qing Hao Miow, Lit-Hsin Loo, Wei Peng Yong, Raghav Sundar

**Affiliations:** 1 Institute of Molecular and Cell Biology, Agency for Science, Technology and Research, Singapore, Singapore; 2 Department of Anatomical Pathology, Singapore General Hospital, Singapore, Singapore; 3 Department of Pathology, National University Health System, Singapore, Singapore; 4 Yong Loo Lin School of Medicine, National University of Singapore, Singapore, Singapore; 5 Biostatistics Unit, Yong Loo Lin School of Medicine, National University of Singapore, Singapore, Singapore; 6 Department of Haematology-Oncology, National University Cancer Institute, Singapore, National University Hospital, Singapore, Singapore; 7 Cancer Science Institute of Singapore, National University of Singapore, Singapore, Singapore; 8 Bioinformatics Institute, Agency for Science, Technology, and Research, Singapore, Singapore; 9 Cancer and Stem Cell Biology Program, Duke-NUS Medical School, Singapore, Singapore; 10 The N.1 Institute for Health, National University of Singapore, Singapore, Singapore; 11 Singapore Gastric Cancer Consortium, Singapore, Singapore

**Keywords:** Biomarkers, Tumor, Immunotherapy, Stomach neoplasms, Immune checkpoint inhibitors

## Abstract

**Background:**

Immune checkpoint inhibitors (ICI) are now standard-of-care treatment for patients with metastatic gastric cancer (GC). To guide patient selection for ICI therapy, programmed death ligand-1 (PD-L1) biomarker expression is routinely assessed via immunohistochemistry (IHC). However, with an increasing number of approved ICIs, each paired with a different PD-L1 antibody IHC assay used in their respective landmark trials, there is an unmet clinical and logistical need for harmonization. We investigated the interchangeability between the Dako 22C3, Dako 28–8 and Ventana SP-142 assays in GC PD-L1 IHC.

**Methods:**

In this cross-sectional study, we scored 362 GC samples for PD-L1 combined positive score (CPS), tumor proportion score (TPS) and immune cells (IC) using a multiplex immunohistochemistry/immunofluorescence technique. Samples were obtained via biopsy or resection of gastric cancer.

**Results:**

The percentage of PD-L1-positive samples at clinically relevant CPS ≥ 1, ≥ 5 and ≥ 10 cut-offs for the 28–8 assay were approximately two-fold higher than that of the 22C3 (CPS ≥ 1: 70.3 vs 49.4%, *p* < 0.001; CPS ≥ 5: 29.1 vs 13.4%, *p* < 0.001; CPS ≥ 10: 13.7 vs 7.0%, *p* = 0.004). The mean CPS score on 28–8 assay was nearly double that of the 22C3 (6.39 ± 14.5 vs 3.46 ± 8.98, *p* < 0.001). At the clinically important CPS ≥ 5 cut-off, there was only moderate concordance between the 22C3 and 28–8 assays.

**Conclusion:**

Our findings suggest that scoring PD-L1 CPS with the 28–8 assay may result in higher PD-L1 scores and higher proportion of PD-L1 positivity compared to 22C3 and other assays. Until stronger evidence of inter-assay concordance is found, we urge caution in treating the assays as equivalent.

**Supplementary Information:**

The online version contains supplementary material available at 10.1007/s10120-022-01301-0.

## Introduction

Immune checkpoint inhibitors targeting the programmed death-1 (PD-1)/programmed death ligand-1 (PD-L1) pathway are now standard of care for patients with various advanced and metastatic cancers, including gastric cancer (GC). Several studies have demonstrated that GC with higher levels of PD-L1 expression tends to derive higher benefit from treatment with anti-PD-1 blockade [1, 2]. Several randomized phase III trials with pembrolizumab (anti-PD-1 antibody) have been designed using the Dako 22C3 assay to select PD-L1 positive populations [3, 4]. This has led to the Food and Drug Administration (FDA) approving pembrolizumab in these specific indications[5] along with the Dako 22C3 assay as a companion diagnostic [6]. Thus, evaluation of PD-L1 expression via immunohistochemistry (IHC) has become an integral part of the treatment algorithm for patients with metastatic gastric cancer.

Presently, various standardized IHC PD-L1 antibody assays (e.g. Dako 22C3, Dako 28–8 and Ventana SP-142) have been approved as companion diagnostics to predict treatment response to different ICIs in various other tumor types (pembrolizumab, nivolumab and atezolizumab) [7]. Each assay requires a different staining protocol, equipment and cut-offs, leading to a potential source of confusion among clinicians and pathologists, and a logistical hassle for laboratories and hospitals. Importantly, CheckMate-649 was a randomized phase III trial that demonstrated the benefit of the addition of nivolumab to chemotherapy in the first-line treatment of metastatic gastric or esophageal adenocarcinoma [8]. The study analyzed various PD-L1 combined positive score (CPS) subgroups as primary and secondary endpoints. While the study demonstrated benefit in the all-randomized CPS ≥ 1 and CPS ≥ 5 populations, there was significant controversy on the benefit in the PD-L1 low expressing population (CPS < 5) [1, 2, 9]. This led to different regulatory approvals in various parts of the world, with the US Food and Drug Administration (FDA) approving nivolumab regardless of CPS score, and the European Medicines Agency (EMA) approving nivolumab only for patients with a PD-L1 IHC score of CPS ≥ 5. To add further complexity, the antibody used to score CPS in CheckMate-649 was the Dako 28–8 antibody [8]. Much uncertainty exists on the concordance of CPS scores between different PD-L1 assays. With an increasing number of approved ICIs and corresponding companion IHC assays [1, 8], there is an unmet clinical need to demonstrate the concordance between these assays to allow interchangeable utilization in the clinic.

## Materials and methods

### Patients and tumors

This is a cross-sectional study using archival formalin-fixed, paraffin-embedded (FFPE) tissue samples from patients obtained via biopsy or resection of gastric cancer at the National University Hospital (NUH), Singapore, between 1997 and 2019. A majority of the samples were developed into a tissue microarray (TMA) while some samples were used as whole-slides for orthogonal validation. All samples selected for the TMA were derived from surgical resection specimens. The samples selected for whole-slide analysis were obtained from gastric cancer patients treated with immune checkpoint inhibitor therapy, and consisted of both surgical resection specimens and biopsies. Cases recorded to be suitable for research and with sufficient tissue for analysis were identified by the Department of Pathology, NUH. Clinicopathological data (including age at diagnosis, gender, ethnicity, disease stage, degree of differentiation and Lauren classification) was annotated for eligible cases, and de-identified. Sections from the tissue samples were prepared and submitted for conventional IHC and multiplex immunohistochemistry/immunofluorescence (mIHC/IF) analysis.

### Patient and public involvement

The National Healthcare Group Domain-Specific Review Board provided ethical approval for the use of patient materials in this study (reference number 2015/00209 and 2020/00189). As this was not an interventional trial, we did not directly involve patients in the design or determination of outcome measures of this study.

### mIHC/IF protocol

**Fig. 1:**
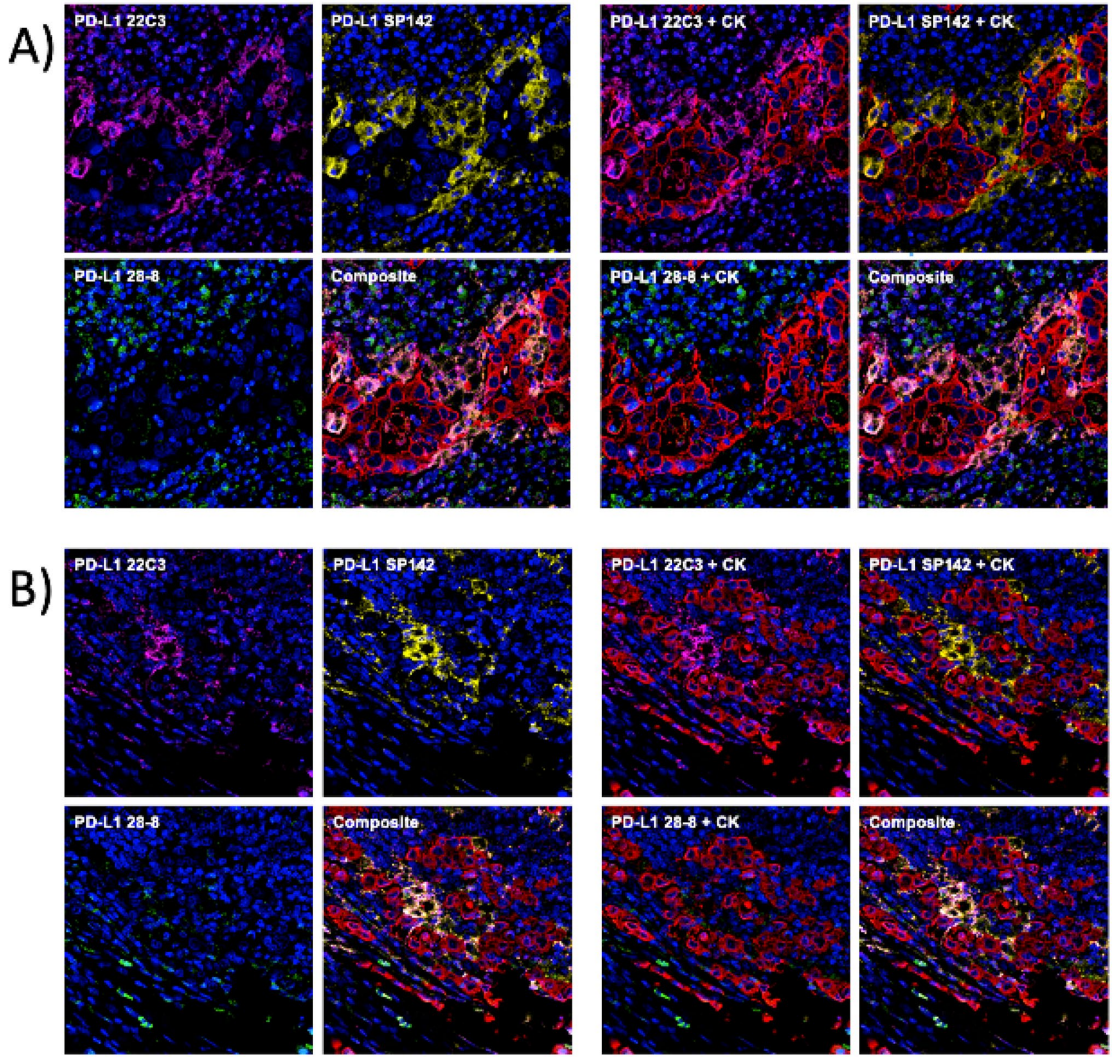
Representative images of gastric cancer tissues stained using multiplex immunohistochemistry/immunofluorescence (mIHC/IF) [DAPI (Blue), PD-L1 22C3 (Magenta), PD-L1 SP142 (Yellow), PD-L1 28–8 (Green), CK (Red)]. (Magnification, 200×), from two different patients (**A**) and (**B**)

mIHC/IF was performed using an Opal Multiplex fIHC kit (Akoya Biosciences, California), as previously described [10–12]. In brief, FFPE tissue sections were cut onto Bond Plus slides (Leica Biosystems, Richmond) and heated at 60 °C for 20 min. The tissue slides were subjected to deparaffinization, rehydration, and heat-induced epitope retrieval using a Leica Bond Max autostainer (Leica Biosystems, Melbourne) before endogenous peroxidase blocking (Leica Biosystems, Newcastle). Next, the slides were incubated with primary antibodies followed by incubation with polymeric HRP-conjugated secondary antibodies (Leica Biosystems, Newcastle) (Fig. 1 and Supplementary Table S2). The samples were incubated with Opal fluorophore-conjugated tyramide signal amplification (TSA) (Akoya Biosciences, California) at 1:100 dilution. The slides were rinsed with wash buffer (BOND Wash Solution 10X Concentrate) after each step. Following TSA deposition, the slides were again subjected to heat-induced epitope retrieval to strip the tissue-bound primary/secondary antibody complexes before further labelling. These steps were repeated until the samples were labelled with all six markers and spectral DAPI (Akoya Biosciences, California) at a 1:10 dilution. Finally, the slides were mounted in ProLong Diamond Anti-fade Mountant (Molecular Probes, Life Technologies, USA) and developed in the dark at room temperature for 24 h. Images were captured for each case under a Vectra 3 pathology imaging system microscope (Akoya Biosciences, California) and then analyzed and scored by a pathologist using inForm software (version 2.4.2; Akoya Biosciences), cellXpress software [13] and HALO™ (Indica Lab). Particularly for PD-L1 scoring formula and algorithm, we followed a detailed protocol that our group previous reported to set up the mIHC/IF-based PD-L1 quantification for multiple PD-L1 simultaneously across cancer types [14].

### CPS, TPS and IC scoring

Consecutive tissue sections from tumors were labelled to allow for the separate quantification of CPS, tumor proportion score (TPS) and immune cells (IC) using the EpCAM-labelled sample as a reference to allow tumor cell counts quantification. CPS was calculated as the number of PD-L1 staining tumor and immune cells divided by the total viable tumor cells multiplied by 100 with a maximum score of 100. TPS was calculated as the percentage of tumor cells showing staining relative to all tumor cells present in the sample. IC was calculated as the proportion of tumor area occupied by PD-L1-positive tumor infiltrating immune cells.

### Analysis for sample-degradation bias

To study the effect of sample-degradation bias, a subgroup analysis was performed, based on the age of the tissue sample. Samples were divided into two cohorts: those obtained in the last 15 years (2007–2021, new cohort) and those obtained more than 15 years ago (2006 and prior, old cohort).

### Statistical analysis

Statistical analyses were performed using R, version 4.0.1 (R Foundation, Vienna, Austria) and SPSS, version 25.0 (IBM Corp., Armonk, United States). Contingency tables and *χ*2 or Fisher’s exact tests (for categorical variables) and *t* test for comparison of means or Mann–Whitney *U* test for comparison of medians (for continuous variables) were used to investigate associations of PD-L1 IHC and mIHC/IF results with clinicopathological characteristics and outcomes. Correlations between the three antibody assays (22C3, SP-142 and 28–8) were analyzed using Spearman’s rank correlations. Spearman’s coefficient varies from 0 (no correlation) to 1 (perfect correlation) [15], with ranges interpreted as follows: 0.01–0.30 (negligible correlation), 0.30–0.50 (weak), 0.51–0.70 (moderate), 0.71–0.90 (strong) and 0.90–1.00 (very strong correlation) [16]. Concordance between the three antibody assays (22C3, SP-142 and 28–8) for each of the scoring systems (CPS, TPS and IC) was assessed using Gwet’s kappa (to account for the paradox of unbalanced main diagonal), with the value of kappa ranging from 0 to 1, with 0 representing total disagreement and 1 total agreement [17]. Values of kappa are interpreted as follows: ≤ 0 (no agreement), 0.01–0.20 (none to slight), 0.21–0.40 (fair), 0.41–0.60 (moderate), 0.61–0.80 (substantial), 0.81–1.00 (almost perfect agreement) [18]. At the same CPS cut-offs, we calculated the classification accuracy, defined as the number of correct predictions made (true positive + true negative) divided by the total number of predictions made. Survival analysis was conducted using the Kaplan–Meier method and compared between different PD-L1 expression groups using a Cox proportional hazards model. For analyses involving pairwise comparison between the antibody assays, the 22C3 assay was used as the reference for comparison with 28–8 and SP-142 assays, respectively. A *p* value < 0.05 was considered to indicate statistical significance unless otherwise stated.

## Results

### Main cohort

#### Patient characteristics

We conducted primary analysis on the main cohort of 344 patients which were analyzed on TMA. Most of the patients were males (236/344, 68.6%) and of Chinese ethnicity (291/344, 85.5%). The median age at diagnosis of the cohort was 68 years (IQR 16.25). Most of the patients had gastric cancer that were poorly differentiated (207/344, 60.2%), and of intestinal subtype based on Lauren classification (173/344, 50.3%). Based on a CPS ≥ 1 on the 22C3 assay, 49.4% of the patients (170/344) were PD-L1 positive. When stratified by PD-L1 positivity status based on CPS ≥ 1 using the 22C3 assay, there was no statistically significant difference in median age or distribution based on gender, ethnicity, stage of disease at diagnosis, degree of differentiation or Lauren classification subtype between both groups (Table 1).

**Table 1:** Patient and sample characteristics stratified by programmed death-ligand 1 (PD-L1) status on multiplex immunohistochemistry using 22C3 assay in the main tissue microarray (TMA) cohort

Variables	CPS < 1 (*n* = 174)	CPS ≥ 1 (*n* = 170)	*p*-value
Median age of patient (IQR)	70 (16)	68 (16)	0.48
Gender			0.75
Male	118 (67.8%)	118 (69.4%)	
Female	56 (32.2%)	52 (30.6%)	
Ethnicity			0.15
Chinese	144 (82.8%)	147 (86.5%)	
Indian	5 (2.9%)	9 (5.3%)	
Malay	8 (4.6%)	7 (4.1%)	
Others	17 (9.8%)	7 (4.1%)	
Stage at diagnosis			0.25
I	30 (17.2%)	38 (22.4%)	
II	37 (21.3%)	27 (15.9%)	
III	89 (51.1%)	80 (47.1%)	
IV	18 (10.3%)	25 (14.7%)	
Differentiation			0.17
Poorly differentiated	103 (59.2%)	104 (61.2%)	
Moderately differentiated	48 (27.6%)	45 (26.5%)	
Well differentiated	3 (1.7%)	9 (5.3%)	
NOS	20 (11.5%)	12 (7.1%)	
Lauren Classification			0.3
Intestinal	91 (52.3%)	82 (48.2%)	
Diffuse	53 (30.5%)	47 (27.6%)	
Mixed	19 (10.9%)	31 (18.2%)	
NOS	11 (6.3%)	10 (5.9%)	
Median Age of Sample in months (IQR)	163 (82.5)	162 (51)	0.53

#### Concordance of PD-L1 status between different assays based on CPS cut-offs

The concordance between the three PD-L1 antibody assays were analyzed at the clinically relevant CPS cut-offs of 1, 5 and 10 [1, 2, 4, 8]. Between the 28–8 and 22C3 assays, scoring with 28–8 assay consistently resulted in a higher proportion of PD-L1 positive samples (70.3 vs 49.4%, *p* < 0.001 at CPS ≥ 1; 29.1 vs 13.4%, *p* < 0.001 at CPS ≥ 5; 13.7 vs 7.0%, *p* = 0.004 at CPS ≥ 10) (Table 2).

**Table 2:** Proportion of PD-L1 positivity with different assays, at CPS cut-offs of 1,5,10 in the main cohort (*n* = 344)

Assay	CPS ≥ 1	CPS ≥ 5	CPS ≥ 10
22C3	170 (49.4%)	46 (13.4%)	24 (7.0%)
28–8	242 (70.3%)	100 (29.1%)	47 (13.7%)
SP-142	170 (49.4%)	68 (19.8%)	33 (9.6%)

At CPS ≥ 1, the classification accuracy between the 22C3 and 28–8 assays was 62.2% with only fair concordance (Gwet’s Kappa = 0.276). Notably, the classification accuracy (73.3 and 85.2%) and concordance (Gwet’s Kappa = 0.598 and 0.818) improved with increasing CPS cut-off of CPS ≥ 5 and CPS ≥ 10, respectively.

The classification accuracy and concordance between the 22C3 and SP142 assays was higher than that between the 22C3 and 28–8 assays at all CPS cut-offs (classification accuracy: 65.2 vs 62.2% at CPS ≥ 1, 80.8 vs 73.3% at CPS ≥ 5, 89.8 vs 85.2% at CPS ≥ 10; Gwet’s Kappa: 0.302 vs 0.276 at CPS ≥ 1, 0.735 vs 0.598 at CPS ≥ 5, 0.880 vs 0.818 at CPS ≥ 10). Results are summarized in Table 3.

**Table 3:** Concordance of PD-L1 status between different assays, at CPS cut-offs of 1,5,10 in the main cohort﻿ (*n* = 344)

CPS cut-offs		22C3 assay	
		CPS < 1	CPS ≥ 1
28–8 assay	CPS < 1	73 (21.2%)	29 (8.4%)
	CPS ≥ 1	101 (29.4%)	141 (41.0%)
	Accuracy	62.2%	
	Gwet’s Kappa	0.276 (*p* < 0.001)	
		CPS < 5	CPS ≥ 5
	CPS < 5	225 (65.4%)	19 (5.5%)
	CPS ≥ 5	73 (21.2%)	27 (7.9%)
	Accuracy	73.3%	
	Gwet’s Kappa	0.598 (*p* < 0.001)	
		CPS < 10	CPS ≥ 10
	CPS < 10	283 (82.3%)	14 (4.1%)
	CPS ≥ 10	37 (10.8%)	10 (2.9%)
	Accuracy	85.2%	
	Gwet’s Kappa	0.818 (*p* < 0.001)	
SP-142 assay		CPS < 1	CPS ≥ 1
	CPS < 1	114 (33.2%)	60 (17.4%)
	CPS ≥ 1	60 (17.4%)	110 (32.0%)
	Accuracy	65.2%	
	Gwet’s Kappa	0.302 (*p* < 0.001)	
		CPS < 5	CPS ≥ 5
	CPS < 5	254 (73.8%)	22(6.4%)
	CPS ≥ 5	44 (12.8%)	24 (7.0%)
	Accuracy	80.8%	
	Gwet’s Kappa	0.735 (*p* < 0.001)	
		CPS < 10	CPS ≥ 10
	CPS < 10	298 (86.6%)	13 (3.8%)
	CPS ≥ 10	22 (6.4%)	11 (3.2%)
	Accuracy	89.8%	
	Gwet’s Kappa	0.880 (*p* < 0.001)	

#### Mean difference in continuous CPS scores obtained from different PD-L1 assays

The mean CPS score obtained from the 28–8 assay was significantly higher than both the 22C3 assay (6.39 ± 14.5 vs 3.46 ± 8.98, *t*(343) = − 4.083, *p* < 0.001) and the SP-142 assay (6.39 ± 14.5 vs 4.08 ± 10.3, *t*(343) = − 3.370, *p* = 0.001).

**Fig. 2:**
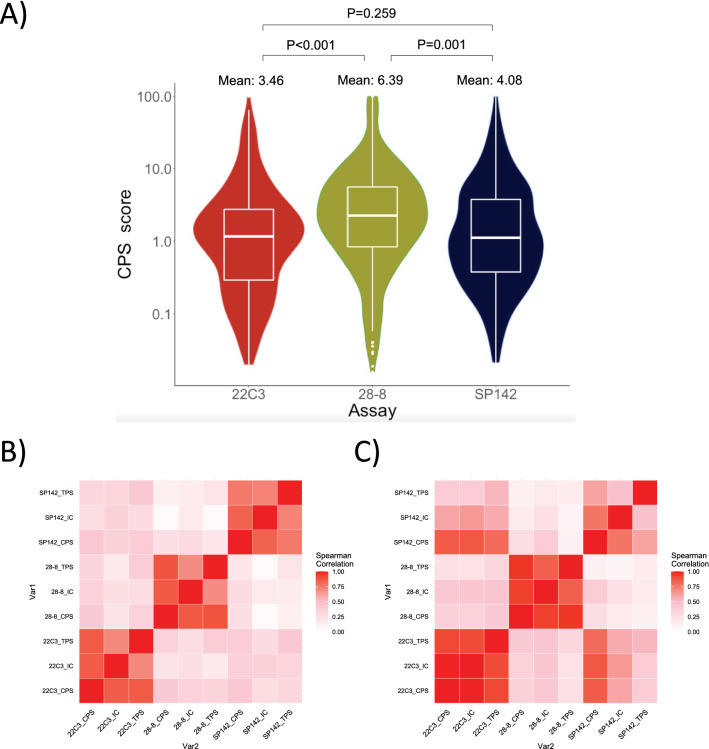
PD-L1 scoring obtained from 22C3, 28 to 8 and SP-142 assays. **A** Log-transformed violin plot of CPS scores on PD-L1 obtained from 22C3, 28 to 8 and SP-142 assays among the main cohort of gastric cancer patients (*n* = 344). **B** Heat map representation of correlation between PD-L1 scoring of CPS, TPS and IC obtained from 22C3, 28 to 8 and SP-142 assays among the main cohort of gastric cancer patients (*n* = 344). **C** Heat map representation of correlation between PD-L1 scoring of CPS, TPS and IC obtained from 22C3, 28 to 8 and SP-142 assays among the additional cohort of ICI-treated gastric cancer patients (*n* = 18)

There was, however, no significant difference between mean CPS scores obtained on 22C3 and SP-142 (3.46 ± 8.98 vs 4.08 ± 10.3, *t*(343) = − 1.130, *p* = 0.259) (Fig. 2A).

#### Correlation between continuous PD-L1 scores obtained from different PD-L1 assays

Between the 28–8 and 22C3 assays, the Spearman’s correlation values were 0.392, 0.381 and 0.230 for CPS, TPS and IC scores, respectively, suggesting consistently weak correlation (Fig. 2B and Supplementary Table S1A).

The correlations between the 28–8 and SP-142 assay were also consistently weak (Spearman’s values of 0.213, 0.180 and 0.133 for CPS, TPS, IC, respectively).

Similar to the trend in concordance analyses, correlations between 22C3 and SP142 were noted to be marginally higher (Spearman’s values of 0.409, 0.417 and 0.347 for CPS, TPS, and IC, respectively).

A subgroup analysis was performed to study the effect of sample-degradation bias found that the inter-assay variability between the various assays persisted across subgroups (Supplementary Tables S3–5), with similar differences in mean CPS scores obtained on the 28–8 and 22C3 assays in both the old (7.46 vs 3.72) and new (5.50 vs 3.23) cohorts, respectively.

### Additional cohort

#### Patient characteristics

In this additional cohort of 18 ICI-treated GC patients (Supplementary Table S6), CPS, TPS and IC scoring with the three assays were performed on whole-slide tissue samples, to compare with the TMA samples. 83.3% of patients (15/18) were treated with nivolumab, and the remaining 16.7% (3/18) with pembrolizumab.

#### Orthogonal validation of PD-L1 scores obtained from different PD-L1 assays

Spearman’s value suggested weak correlation between the 22C3 and 28–8 assays, similar to the trend observed in the main cohort. (Fig. 2C and Supplementary Table S1B).

Additionally, we assessed the correlation between PD-L1 scores from the three antibody assays in a combined analysis comprising both the main and additional cohort (Supplementary Table S1C). The correlations between CPS scores obtained on the 22C3 and 28–8 assays were similar across all three analyses (TMA only = 0.392, whole-slide only = 0.360, TMA and whole-slide combined = 0.414) (Supplementary Tables S1A–S1C). In this cohort, 11 (61.1%) samples were derived from endoscopic biopsy while the rest were derived from surgical resection. The proportion of PD-L1 positivity did not differ significantly between biopsy and resected specimens (Supplementary Table S7).

#### Correlation between CPS scores and survival with immunotherapy treatment

In the main TMA cohort, CPS positivity status did not guide the treatment modality as this patient database predated the advent of mainstream immunotherapy in gastric adenocarcinoma treatment. Therefore, to assess any relation between PD-L1 status and survival, we also performed an exploratory survival analysis in this additional cohort of 18 ICI-treated patients.

In these patients treated with immunotherapy, though statistical significance was not observed in this small cohort, univariate Cox regression demonstrated a trend toward improved overall survival in PD-L1-positive patients at the CPS cut-off of ≥ 5 on the 22C3 and 28–8 assays. (Supplementary Figure S1 and Supplementary Table S8).

## Discussion

To address the interchangeability of the various commercially available assays, we performed one of the largest comparisons of PD-L1 assays in gastric adenocarcinoma with more than 350 samples. To our knowledge, our study is the first large-scale study to harness mIHC to comprehensively score PD-L1 expression on a single slide and assess the interchangeability of the different PD-L1 assays in a gastric cancer dataset. Given the spatial heterogeneity of gastric cancer [19], there are advantages of performing mIHC on a single slide compared to conventional IHC on consecutive slides to study the various PD-L1 assays.

One of the first FDA approvals for ICI in GC was pembrolizumab as third-line treatment for GC patients with CPS ≥ 1. This was based on the results of the KEYNOTE-059 study, with the 22C3 assay approved as the companion diagnostic [2]. More recently, the EMA approved nivolumab in combination with chemotherapy for the first-line treatment of metastatic GC in patients with CPS ≥ 5. This approval was based on the results of the CheckMate 649 study [8], which utilized the Dako 28–8 assay. As pathology labs across the globe were already performing GC PD-L1 CPS score using the 22C3 assay, we paid particular attention to the inter-assay concordance at CPS ≥ 5 between 22C3 and 28–8 [20]. Considering the significantly higher PD-L1 positivity rate using 28–8 compared to 22C3 especially at CPS ≥ 5, our findings suggest that using the 22C3 assay in lieu of the 28–8 assay will likely result in fewer patients eligible for first-line nivolumab if prescribed as per EMA indication. However, it must be noted that there remains significant controversy on the use of ICI in PD-L1 low expressing tumors (CPS < 10), and the clinical impact of this difference between assays needs to be established [9]. This is probably best done by performing both the 22C3 and 28–8 assays in the pivotal trials that have been conducted such as CheckMate-649 or KEYNOTE-590, a trial which led to the indication of first-line pembrolizumab in esophageal cancer in CPS ≥ 10, independent of histology and, therefore, includes adenocarcinomas of the gastro-esophageal junction [21].

This variability in PD-L1 scores observed between the different assays may thus present a dilemma for clinicians in determining patient eligibility for ICI therapy. This is confounded by the difference in the IHC antibody assays used in landmark clinical trials. Notably, trials utilizing different PD-L1 assays have reported PD-L1 positivity proportions that are remarkably consistent with our study. The CheckMate 649 study, which used the 28–8 assay, reported double the prevalence (60%) of patients with CPS ≥ 5, compared to the KEYNOTE-061 study (31%), which utilized the 22C3 assay [2, 8].

Furthermore, in a recent smaller study by Ahn et al*.* (*n* = 55)*,* though the 22C3 and 28–8 assays were found to be comparable at various CPS cut-offs, they similarly reported that the CPS scores on 28–8 assay was more frequently higher than that of the 22C3 assay, than vice versa (29% vs 4%) and the 28–8 assay more often detected PD-L1 expression in immune cells [7]. While other factors such as heterogeneity in patient population and tumor characteristics may also play a role [22, 23], this phenomenon is likely explained by the analytical discordance between the various assays, especially since similar observations were made in other tumor types with the same antibody assays [24, 25].

Overall, these findings hence do not support the interchangeability of the assays in determining the PD-L1 status of gastric adenocarcinoma. Despite the logistical concerns associated with conducting different assays on the same patient, it may still be necessary to use the distinct assays as companion diagnostics to predict treatment response to their respective ICIs.

Some limitations of our study must be acknowledged. First, our main cohort mIHC analyses were conducted using TMA instead of whole slides. There may be concern that the small tumor area technique may not capture the true heterogeneity in biomarker expression as accurately as larger volume samples [26]. To mitigate this limitation, we included a second cohort of whole-slide mIHC analysis as an orthogonal validation, which reaffirmed the comparability of TMA and whole-slide analyses in our study. Second, marker intensity measured via mIHC may be less robust compared to conventional IHC [27]. These drawbacks can be mitigated by a number of steps during mIHC use, including (1) the confirmation of tumor classification activity, (2) the exclusion of abundant macrophage presence, (3) avoidance of slides with large areas of spurious staining, and (4) confirmation of lower threshold sensitivity levels [28]. This is consistent with our group’s previous demonstration that PD-L1 scoring results obtained from mIHC are concordant to those obtained through conventional IHC methods [14]. Therefore, it is unlikely for the use of mIHC to have substantially biased our results. The age of tissue sample may play a role in sample-degradation bias. A study has previously reported that PD-L1 expression in freshly obtained biopsies (≤ 42 days) had higher positivity rates compared to older samples, in particular those > 900 days [29]. As the samples from our cohort were of varying ages, we performed a subgroup analysis, based on the age of the samples and confirmed that the inter-assay variability between the 22C3 and 28–8 assays persisted across subgroups. Further, the frequency of PD-L1 positivity in our cohort is not dissimilar to those reported in other studies and trials (CPS ≥ 1: our cohort 49 to 70%, CheckMate-649 [8] 60%, Ahn et al. [7] 46 to 49%). However, as CheckMate-649 was a first-line study, it is likely the samples analyzed were freshly obtained which might explain the slightly higher PD-L1 positivity rates. Finally, inter-assay concordance may not necessarily translate to equivalent survival outcomes with immunotherapy treatment—this should be investigated in future cohorts.

## Conclusion

In a large cohort of gastric adenocarcinoma patients, the percentage of PD-L1-positive samples at various CPS cut-offs for the 28–8 assay were approximately twofold higher than that of the 22C3 assay, with only moderate concordance between the 22C3 and 28–8 assays at CPS ≥ 5. These findings do not support the interchangeability of the assays for determining the PD-L1 status of gastric adenocarcinoma, at the clinically relevant CPS cut-off of ≥ 5. Until stronger evidence of inter-assay concordance is found, we urge caution in treating the various assays as equivalent.

## Supplementary Information

Below is the link to the electronic supplementary material.

Supplementary file1 (DOCX 66 KB)
