# Supplementary material for: Choice of PD-L1 immunohistochemistry assay influences clinical eligibility for gastric cancer immunotherapy
Source: Gastric Cancer. 2022 Jun 4;25(4):741–50. doi: 10.1007/s10120-022-01301-0 (PMC9226082; doi:10.1007/s10120-022-01301-0)
Supplement: Supplementary file 1 — Supplementary file1 (DOCX 66 KB) [file 10120_2022_1301_MOESM1_ESM.docx]

**SUPPLEMENTARY MATERIAL**

**Supplementary Table S1A: Spearman’s rank-order correlation between the between PD-L1 scoring of CPS, TPS and IC obtained from 22C3, 28-8 and SP-142 assays among the main cohort of TMA samples**

|  | 22C3_TPS | 22C3_CPS | 22C3_IC | SP-142_TPS | SP-142_CPS | SP-142_IC | 28-8_TPS | 28-8_CPS | 28-8_IC |
| --- | --- | --- | --- | --- | --- | --- | --- | --- | --- |
| 22C3_TPS | 1.000 | .857 | .692 | .417 | .380 | .244 | .381 | .326 | .241 |
| 22C3_CPS | .857 | 1.000 | .843 | .306 | .409 | .230 | .319 | .392 | .331 |
| 22C3_IC | .692 | .843 | 1.000 | .304 | .338 | .347 | .139 | .176 | .230 |
| SP-142_TPS | .417 | .306 | .304 | 1.000 | .740 | .710 | .180 | 0.090 | .134 |
| SP-142_CPS | .380 | .409 | .338 | .740 | 1.000 | .815 | .147 | .213 | .249 |
| SP-142_IC | .244 | .230 | .347 | .710 | .815 | 1.000 | 0.003 | 0.002 | .133 |
| 28-8_TPS | .381 | .319 | .139 | .180 | .147 | 0.003 | 1.000 | .871 | .678 |
| 28-8_CPS | .326 | .392 | .176 | 0.090 | .213 | 0.002 | .871 | 1.000 | .858 |
| 28-8_IC | .241 | .331 | .230 | .134 | .249 | .133 | .678 | .858 | 1.000 |

**Supplementary Table S1B: Spearman’s rank-order correlation between the between PD-L1 scoring of CPS, TPS and IC obtained from 22C3, 28-8 and SP-142 assays among the additional cohort of whole-slide samples**

|  | 22C3_TPS | 22C3_CPS | 22C3_IC | SP-142_TPS | SP-142_CPS | SP-142_IC | 28-8_TPS | 28-8_CPS | 28-8_IC |
| --- | --- | --- | --- | --- | --- | --- | --- | --- | --- |
| 22C3_TPS | 1.000 | .911 | .905 | .529 | .800 | .573 | 0.329 | 0.414 | 0.455 |
| 22C3_CPS | .911 | 1.000 | .99 | 0.393 | .851 | .598 | 0.236 | 0.360 | 0.428 |
| 22C3_IC | .905 | .992 | 1.000 | 0.389 | .866 | .641 | 0.230 | 0.358 | 0.441 |
| SP-142_TPS | .529 | 0.393 | 0.389 | 1.000 | .604 | .486 | 0.160 | 0.127 | 0.164 |
| SP-142_CPS | .800 | .851 | .866 | .604 | 1.000 | .773 | 0.115 | 0.261 | 0.397 |
| SP-142_IC | .573 | .598 | .641 | .48 | .773 | 1.000 | 0.086 | 0.162 | 0.238 |
| 28-8_TPS | 0.329 | 0.236 | 0.230 | 0.160 | 0.115 | 0.086 | 1.000 | .961 | .841 |
| 28-8_CPS | 0.414 | 0.360 | 0.358 | 0.127 | 0.261 | 0.162 | .961 | 1.000 | .936 |
| 28-8_IC | 0.455 | 0.428 | 0.441 | 0.164 | 0.397 | 0.238 | .841 | .936 | 1.000 |

**Supplementary Table S1C: Spearman’s rank-order correlation between the between PD-L1 scoring of CPS, TPS and IC obtained from 22C3, 28-8 and SP-142 assays among the entire cohort of both TMA and whole-slide samples**

|  | 22C3_TPS | 22C3_CPS | 22C3_IC | SP-142_TPS | SP-142_CPS | SP-142_IC | 28-8_TPS | 28-8_CPS | 28-8_IC |
| --- | --- | --- | --- | --- | --- | --- | --- | --- | --- |
| 22C3_TPS | 1.000 | 0.860 | 0.705 | 0.433 | 0.399 | 0.268 | 0.399 | 0.349 | 0.261 |
| 22C3_CPS | 0.860 | 1.000 | 0.854 | 0.330 | 0.434 | 0.262 | 0.346 | 0.414 | 0.349 |
| 22C3_IC | 0.705 | 0.854 | 1.000 | 0.330 | 0.373 | 0.376 | 0.178 | 0.211 | 0.256 |
| SP-142_TPS | 0.433 | 0.330 | 0.330 | 1.000 | 0.753 | 0.725 | 0.208 | 0.125 | 0.163 |
| SP-142_CPS | 0.399 | 0.434 | 0.373 | 0.753 | 1.000 | 0.830 | 0.192 | 0.250 | 0.279 |
| SP-142_IC | 0.268 | 0.262 | 0.376 | 0.725 | 0.830 | 1.000 | 0.050 | 0.047 | 0.166 |
| 28-8_TPS | 0.399 | 0.346 | 0.178 | 0.208 | 0.192 | 0.050 | 1.000 | 0.879 | 0.691 |
| 28-8_CPS | 0.349 | 0.414 | 0.211 | 0.125 | 0.250 | 0.047 | 0.879 | 1.000 | 0.864 |
| 28-8_IC | 0.261 | 0.349 | 0.256 | 0.163 | 0.279 | 0.166 | 0.691 | 0.864 | 1.000 |

**Supplementary Table S1D: Spearman’s rank-order correlation between the between PD-L1 scoring of CPS, TPS and IC obtained from 22C3, 28-8 and SP-142 assays among the whole slide samples obtained via biopsy**

|  | 22C3_TPS | 22C3_CPS | 22C3_IC | SP-142_TPS | SP-142_CPS | SP-142_IC | 28-8_TPS | 28-8_CPS | 28-8_IC |
| --- | --- | --- | --- | --- | --- | --- | --- | --- | --- |
| 22C3_TPS | 1.000 | 0.891 | 0.873 | 0.436 | 0.800 | 0.436 | 0.227 | 0.282 | 0.227 |
| 22C3_CPS | 0.891 | 1.000 | 0.991 | 0.209 | 0.918 | 0.636 | 0.209 | 0.264 | 0.273 |
| 22C3_IC | 0.873 | 0.991 | 1.000 | 0.164 | 0.909 | 0.682 | 0.245 | 0.291 | 0.327 |
| SP-142_TPS | 0.436 | 0.209 | 0.164 | 1.000 | 0.227 | 0.182 | 0.018 | -0.164 | -0.300 |
| SP-142_CPS | 0.800 | 0.918 | 0.909 | 0.227 | 1.000 | 0.655 | 0.136 | 0.227 | 0.318 |
| SP-142_IC | 0.436 | 0.636 | 0.682 | 0.182 | 0.655 | 1.000 | 0.464 | 0.400 | 0.445 |
| 28-8_TPS | 0.227 | 0.209 | 0.245 | 0.018 | 0.136 | 0.464 | 1.000 | 0.936 | 0.791 |
| 28-8_CPS | 0.282 | 0.264 | 0.291 | -0.164 | 0.227 | 0.400 | 0.936 | 1.000 | 0.909 |
| 28-8_IC | 0.227 | 0.273 | 0.327 | -0.300 | 0.318 | 0.445 | 0.791 | 0.909 | 1.000 |

**Supplementary Table S1E: Spearman’s rank-order correlation between the between PD-L1 scoring of CPS, TPS and IC obtained from 22C3, 28-8 and SP-142 assays among the whole slide samples obtained via resection**

|  | 22C3_TPS | 22C3_CPS | 22C3_IC | SP-142_TPS | SP-142_CPS | SP-142_IC | 28-8_TPS | 28-8_CPS | 28-8_IC |
| --- | --- | --- | --- | --- | --- | --- | --- | --- | --- |
| 22C3_TPS | 1.000 | 0.857 | 0.893 | 0.214 | 0.429 | 0.393 | 0.286 | 0.429 | 0.321 |
| 22C3_CPS | 0.857 | 1.000 | 0.964 | 0.357 | 0.500 | 0.536 | 0.214 | 0.357 | 0.107 |
| 22C3_IC | 0.893 | 0.964 | 1.000 | 0.429 | 0.607 | 0.607 | 0.143 | 0.321 | 0.179 |
| SP-142_TPS | 0.214 | 0.357 | 0.429 | 1.000 | 0.929 | 0.750 | 0.321 | 0.357 | 0.321 |
| SP-142_CPS | 0.429 | 0.500 | 0.607 | 0.929 | 1.000 | 0.893 | 0.107 | 0.179 | 0.179 |
| SP-142_IC | 0.393 | 0.536 | 0.607 | 0.750 | 0.893 | 1.000 | -0.143 | -0.107 | -0.071 |
| 28-8_TPS | 0.286 | 0.214 | 0.143 | 0.321 | 0.107 | -0.143 | 1.000 | 0.964 | 0.821 |
| 28-8_CPS | 0.429 | 0.357 | 0.321 | 0.357 | 0.179 | -0.107 | 0.964 | 1.000 | 0.857 |
| 28-8_IC | 0.321 | 0.107 | 0.179 | 0.321 | 0.179 | -0.071 | 0.821 | 0.857 | 1.000 |

**Supplementary Table S2. Antibodies used for multiplex immunohistochemistry**

| **Primary antibody** | **Company** | **Clone** |
| --- | --- | --- |
| EpCAM | Biolegend | 9C4 |
| Pan-CK | Dako/ Agilent | AE1/AE3 |
| PD-L1 | Dako/ Agilent | 22C3 |
| PD-L1 | Ventana/ Roche Diagnostics | SP142 |
| PD-L1 | Dako/ Agilent | 28-8 |
| CD45 | Dako/ Agilent | M0701 |

**Supplementary Table S3: Concordance of PD-L1 status between different assays, at CPS cut-offs of 1,5,10, grouped by age of the sample**

**Samples from the old cohort (1999-2006) (n=156):**

| CPS cut-offs | | 22C3 assay | |
| --- | --- | --- | --- |
|  |  | **CPS <1** | **CPS ≥1** |
| 28-8 assay | **CPS <1** | 36 (23.1%) | 13 (8.3%) |
|  | **CPS ≥1** | 55 (35.3%) | 52 (33.3%) |
|  | Accuracy | 56.4% | |
|  | Gwet’s Kappa | 0.137(p =0.09) | |
|  |  | **CPS <5** | **CPS ≥5** |
|  | **CPS <5** | 104 (66.7%) | 8 (5.1%) |
|  | **CPS ≥5** | 31 (19.9%) | 13 (8.3%) |
|  | Accuracy | 75.0% | |
|  | Gwet’s Kappa | 0.626 (p <0.001) | |
|  |  | **CPS <10** | **CPS ≥10** |
|  | **CPS <10** | 128 (82.1%) | 4 (2.6%) |
|  | **CPS ≥10** | 18 (11.5%) | 6 (3.8%) |
|  | Accuracy | 85.9% | |
|  | Gwet’s Kappa | 0.825 (p <0.001) | |
| SP-142 assay |  | **CPS <1** | **CPS ≥1** |
|  | **CPS <1** | 56 (35.9%) | 13 (8.3%) |
|  | **CPS ≥1** | 35 (22.4%) | 52 (33.3%) |
|  | Accuracy | 80.4% | |
|  | Gwet’s Kappa | 0.650 (p <0.001) | |
|  |  | **CPS <5** | **CPS ≥5** |
|  | **CPS <5** | 108 (69.2%) | 7 (4.5%) |
|  | **CPS ≥5** | 27 (17.3%) | 14 (9.0%) |
|  | Accuracy | 78.2% | |
|  | Gwet’s Kappa | 0.680 (p <0.001) | |
|  |  | **CPS <10** | **CPS ≥10** |
|  | **CPS <10** | 133 (85.3%) | 3 (1.9%) |
|  | **CPS ≥10** | 13 (8.3%) | 7 (4.5%) |
|  | Accuracy | 89.7% | |
|  | Gwet’s Kappa | 0.876 (p <0.001) | |

**Samples from the new cohort (2007-2013) (n=188):**

| CPS cut-offs | | 22C3 assay | |
| --- | --- | --- | --- |
|  |  | **CPS <1** | **CPS ≥1** |
| 28-8 assay | **CPS <1** | 37 (19.7%) | 16 (8.5%) |
|  | **CPS ≥1** | 46 (24.5%) | 89 (47.3%) |
|  | Accuracy | 67.0% | |
|  | Gwet’s Kappa | 0.387 (p <0.001) | |
|  |  | **CPS <5** | **CPS ≥5** |
|  | **CPS <5** | 121 (64.4%) | 11 (5.9%) |
|  | **CPS ≥5** | 42 (22.3%) | 14 (7.4%) |
|  | Accuracy | 71.8% | |
|  | Gwet’s Kappa | 0.574 (p <0.001) | |
|  |  | **CPS <10** | **CPS ≥10** |
|  | **CPS <10** | 155 (82.4%) | 10 (5.3%) |
|  | **CPS ≥10** | 19 (10.1%) | 4 (2.1%) |
|  | Accuracy | 84.6% | |
|  | Gwet’s Kappa | 0.812 (p <0.001) | |
| SP-142 assay |  | **CPS <1** | **CPS ≥1** |
|  | **CPS <1** | 58 (30.9%) | 47 (25.0%) |
|  | **CPS ≥1** | 25 (13.3%) | 58 (30.9%) |
|  | Accuracy | 61.7% | |
|  | Gwet’s Kappa | 0.235 (p =0.001) | |
|  |  | **CPS <5** | **CPS ≥5** |
|  | **CPS <5** | 146 (77.7%) | 15 (8.0%) |
|  | **CPS ≥5** | 17 (9.0%) | 10 (5.3%) |
|  | Accuracy | 83.0% | |
|  | Gwet’s Kappa | 0.777 (p <0.001) | |
|  |  | **CPS <10** | **CPS ≥10** |
|  | **CPS <10** | 165 (87.8%) | 10 (5.3%) |
|  | **CPS ≥10** | 9 (4.8%) | 4 (2.1%) |
|  | Accuracy | 89.9% | |
|  | Gwet’s Kappa | 0.883 (p <0.001) | |

**Supplementary Table S4: Proportion of PD-L1 positivity with different assays, at CPS cut-offs of 1,5,10, grouped by age of the sample**

**Samples from the old cohort (1999-2006) (n=156):**

| Assay | CPS ≥1 | CPS ≥5 | CPS ≥10 |
| --- | --- | --- | --- |
| 22C3 | 65 (41.6%) | 21 (13.5%) | 10 (6.4%) |
| 28-8 | 107 (68.6%) | 44 (28.2%) | 24 (15.4%) |
| SP-142 | 87 (55.8%) | 41 (26.3%) | 20 (12.8%) |

**Samples from the new cohort (2007-2013) (n=188):**

| Assay | CPS ≥1 | CPS ≥5 | CPS ≥10 |
| --- | --- | --- | --- |
| 22C3 | 105 (55.9%) | 25 (13.3%) | 14 (7.4%) |
| 28-8 | 135 (71.8%) | 56 (29.8%) | 23 (12.2%) |
| SP-142 | 83 (44.1%) | 27 (14.4%) | 13 (69.1%) |

**Supplementary Table S5A: Mean CPS scores on the 22C3, 28-8 and SP142 assays, grouped by age of the sample**

| **Assay** | **Cohort** | **Mean** |
| --- | --- | --- |
| 22C3 | Old cohort (1999-2006) | 3.72±11.2 |
| 28-8 |  | 7.46±17.6 |
| SP142 |  | 5.66±13.8 |
| 22C3 | New cohort (2007-2013) | 3.23±6.55 |
| 28-8 |  | 5.50±11.2 |
| SP142 |  | 2.76±5.69 |

**Supplementary Table S5B: Paired t-tests of mean CPS scores between the 22C3, 28-8 and SP142**

**assays, grouped by age of the sample**

| **Old cohort (1999-2006)** | |
| --- | --- |
|  | **22C3** |
| **28-8** | t(155)=-3.000, p=0.003 |
| **SP142** | t(155)=-1.916, p=0.057 |
| **New cohort (2007-2013)** | |
|  | **22C3** |
| **28-8** | t(187)=-4.083, p=0.006 |
| **SP142** | t(187)=-0.896, p=0.371 |

**Supplementary Table S6: Patient and sample characteristics in the additional whole-slide cohort.**

| **Median age (IQR)** | 62.5 (21.5) |
| --- | --- |
| **Gender**   - Male - Female | 12 (66.7%)  6 (33.3%) |
| **Ethnicity**   - Chinese - Indian - Malay - Others | 13 (72.2%)  2 (11.1%)  1 (5.6%)  2 (11.1%) |
| **Stage at diagnosis**   - I - II - III - IV | 0 (0.0%)  6 (33.3%)  3 (16.7%)  9 (50.0%) |
| **Differentiation**   - Poorly differentiated - Moderately differentiated - Well differentiated - NOS | 13 (72.2%)  5 (27.8%)  0 (0.0%)  0 (0.0%) |
| **Lauren Classification**   - Intestinal - Diffuse - Mixed - NOS | 8 (44.4%)  8 (44.4%)  2 (11.1%)  0 (0.0%) |

**Supplementary Table S7: Proportion of PD-L1 positivity in the additional cohort with different assays, at CPS cut-offs of 1,5,10, grouped by sampling method**

**Samples obtained via biopsy (n=11):**

| Assay | CPS ≥1 | CPS ≥5 | CPS ≥10 |
| --- | --- | --- | --- |
| 22C3 | 9 (81.8%) | 5 (45.5%) | 3 (27.3%) |
| 28-8 | 10 (90.9%) | 8 (72.7%) | 4 (36.4%) |
| SP-142 | 11 (100%) | 9 (81.8%) | 4 (36.4%) |

**Samples obtained via resection (n=7):**

| Assay | CPS ≥1 | CPS ≥5 | CPS ≥10 |
| --- | --- | --- | --- |
| 22C3 | 5 (71.4%) | 1 (14%) | 1 (14%) |
| 28-8 | 7 (100%) | 4 (57.1%) | 3 (42.9%) |
| SP-142 | 6 (85.7%) | 3 (42.9%) | 1 (14%) |

**Supplementary Table S8:** **Analysis of overall survival with cox proportional hazards regression in the additional cohort of 18 patients based on CPS status using different antibody assays.**

|  | CPS >1 | | | | CPS >5 | | | | CPS >10 | | | |
| --- | --- | --- | --- | --- | --- | --- | --- | --- | --- | --- | --- | --- |
|  | HR | 95% CI lower | 95% CI upper | p | HR | 95 CI % lower | 95CI % upper | p | HR | 95 CI % lower | 95 CI % upper | p |
| 22C3 | 1.720 | 0.4665 | 6.341 | 0.415 | 0.625 | 0.172 | 2.273 | 0.476 | 1.072 | 0.2258 | 5.091 | 0.930 |
| 28-8 | 7.583e-10 | 0 | Inf | 0.998 | 0.556 | 0.1849 | 1.673 | 0.297 | 0.458 | 0.1392 | 1.509 | 0.199 |
| SP-142 | 3.004e+08 | 0 | Inf | 0.998 | 1.485 | 0.476 | 4.63 | 0.496 | 0.816 | 0.2529 | 2.633 | 0.734 |

**Supplementary Figure S1: Kaplan-Meier analysis of overall survival in the additional cohort of 18 patients based on CPS status using different antibody assays.**

| 22C3 CPS≥5   | 28-8 CPS≥5   |
| --- | --- |

**Representative images of gastric cancer tissue stained using multiplex immunohistochemistry/ immunofluorescence (mIHC/IF)**

Additional images are available at <https://immunoatlas.org/ATLA/220423-1/>
